# Getting clear about the F-word in genomics

**DOI:** 10.1371/journal.pgen.1008702

**Published:** 2020-04-01

**Authors:** Stefan Linquist, W. Ford Doolittle, Alexander F. Palazzo

**Affiliations:** 1 Department of Philosophy, University of Guelph, Guelph, Ontario, Canada; 2 Department of Biochemistry and Molecular Biology, Dalhousie University, Halifax, Nova Scotia, Canada; 3 Department of Biochemistry, University of Toronto, Toronto, Ontario, Canada; University of California Los Angeles, UNITED STATES

Although biology is generally awash with adaptationist “just-so” stories, the situation in molecular biology and genomics is particularly bad. Various types of non-coding DNA are routinely interpreted as functional without adequate consideration of non-adaptationist alternative hypotheses [1]. Part of the problem is surely due to a failure in these disciplines to appreciate theoretical developments in population genetics, which outline the conditions under which genetic elements are selected [2]. However, as a number of authors have noted, the problem is also partly due to a confusion about the various possible meanings of “function” in biology [3–5]. Our central thesis is that there exists an overlooked dichotomy in the way that researchers see natural selection to be related to function. Traits or genetic elements that are merely under purifying selection have what we call *maintenance functions* whereas those that have historically been under directional selection have *origin functions*. We argue that ignoring this distinction encourages a form of pan-adaptationism, where highly plausible non-adaptive explanations for the origins of certain genetic elements or traits are themselves ignored. Thus, our recommendation is for researchers to always clarify which sense of “function” they mean (origin or maintenance) when talking or writing about selected effects.

Before developing this argument, it is important to clarify our position by distinguishing selection-based notions of functions as a class from causal role (CR) functions. Although this distinction is widely recognized by philosophers of biology our sense is that it remains unfamiliar to many biologists. The CR definition of function is extremely permissive. It applies to any of the effects which a component has on the system(s) that contain it, irrespective of their impact (or that system’s impact) on fitness. For example, a mobile genetic element which elevates mutation rate in the genome has this effect as one of its CR functions, even if it causes a net decrease in organismal fitness. Such permissiveness in the definition of CR function has led some researchers to dismiss this concept [6]. This reaction is understandable when it comes from researchers working in the disciplines of ecology or evolution, where there is often an emphasis on the ecological roles performed by a given trait and their effects on organismal fitness. More controversial is whether researchers working in molecular biology or bioinformatics would embrace the CR concept once its commitment to fitness neutrality is made explicit. On the one hand, investigators in these disciplines might point out that they use methods (e.g. biochemical interaction measurements) that can only establish an entity’s causal roles. To infer a contribution to fitness (and thus selection) requires an additional and difficult-to-prove inference, namely that those causal role “functions” have indeed been under selection. As it turns out, sometimes those inferences are poorly supported—as in the publicity surrounding ENCODE, which we discuss below. Nonetheless, from this perspective it makes sense to view much of the work in molecular biology or bioinformatics as being focussed primarily on CR functions.

Despite its practicality, there is a problem with this line of reasoning. Although molecular biologists rarely measure and compare the relative fitness values of genetic variants, their investigations into molecular mechanisms are nonetheless steeped in evolutionary considerations. Hence, most researchers are in fact searching for entities which they presume to have been under some form of selection. This assumption is so foundational in the fields of molecular biology and genomics that it is rarely stated explicitly in most publications. However, if ideas about selection and fitness are influencing (even implicitly) researchers’ choices about which mechanisms to functionally investigate, then it follows that those researchers are not in fact operating with a CR definition of function, because CR functions are by definition insensitive to such considerations [7].

Of course, it is a separate question whether researchers tend to overestimate the extent to which selection has shaped those entities. There is a tendency among molecular biologists and bioinformaticians to assume by default that if some structure or process exists, it has probably been under purifying selection [8]. For example, untranslated regions of mRNAs and certain ncRNAs have been widely assumed to have some impact on fitness simply because they are made. Their low level of sequence conservation has been brushed aside by some, with the idea that RNA molecules that play a role in cell physiology can tolerate many more mutations than coding-RNA [9,10]. Although there is evidence that some of these RNAs have indeed been under purifying selection to eliminate insertions and deletions [11], the appropriate null hypothesis in such cases is that such entities have no impact on fitness [12]. Indeed, the distinction we shall now develop is intended to encourage researchers to be more cautious about drawing functional inferences from ambiguous data.

Our primary focus is on what are called selected effect (SE) functions and the processes that generate or maintain them. The SE function of some gene or trait is usually defined as the role for which it has been “under selection”. However, this definition fails to distinguish between two distinct processes. On the one hand, to say that an entity is under selection might simply mean that it has recently been subject to purifying selection. Purifying selection occurs whenever heritable modifications to an entity tend to decrease fitness and are thus removed or “purified” from the population. Perhaps the simplest examples are “essential genes” whose deletion is lethal, or highly conserved amino acids in the sequence of the encoded protein. Purifying selection causes these entities to remain relatively static or unchanged over evolutionary time and this is explained by the fact that they have some fitness-contributing effect. Evidence in favour of such selection would be evolutionary conservation: presence of the element or sequence across taxa. By contrast, sequences or traits that are not under purifying selection might undergo significant modifications without deleterious effects on fitness. Such “currently dispensable” items are in an important sense non-functional regardless of whether they originated by neutral evolution or previously had some selected effect that is no longer under purifying selection (i.e. have become a relic, as with the appendix).

Alternatively, we might also contemplate a scenario in which a gene or trait acquires its current function by a process of adaptation. In this scenario, an entity that is either created or modified by mutation undergoes positive selection, and through several rounds of mutation and positive selection it is molded to perform its current role. Philosophers of biology often treat selected effect (SE) functions as if all were adaptations, the underlying traits and the genes encoding them once having increased (and possibly still increasing) in frequency in the relevant population due to “positive” or “directional” selection [13,14]. Biologists too, often do this, focusing on complex adaptations that have undergone improvement in performance as a result of many successive rounds of variation and positive selection. Our favorite example comes from John Maynard Smith, focusing on hearts, also a favorite organ among philosophers [15].

“…If we say that the function of the heart is to pump blood round the body, we do not mean merely that the heart does, as a matter of fact, pump blood. We mean that the heart evolved because it pumped blood; that is, those animals whose hearts were better pumps survived and left more descendants…”

Included as adaptations, we think, would be traits that arose through selection for one effect but are now under directional positive selection for another role–one sense to which Gould and Vrba applied the term ***exaptation*** [16]. We would like to differentiate all such adaptations from entities that are being maintained by purifying selection, but which never experienced directional or positive selection for the effect now maintained (or possibly any effect). As we now explain, this includes the rest of what Gould and Vrba called exaptations and the dependencies produced by “Constructive Neutral Evolution” (CNE [17,18]).

**Fig 1:**
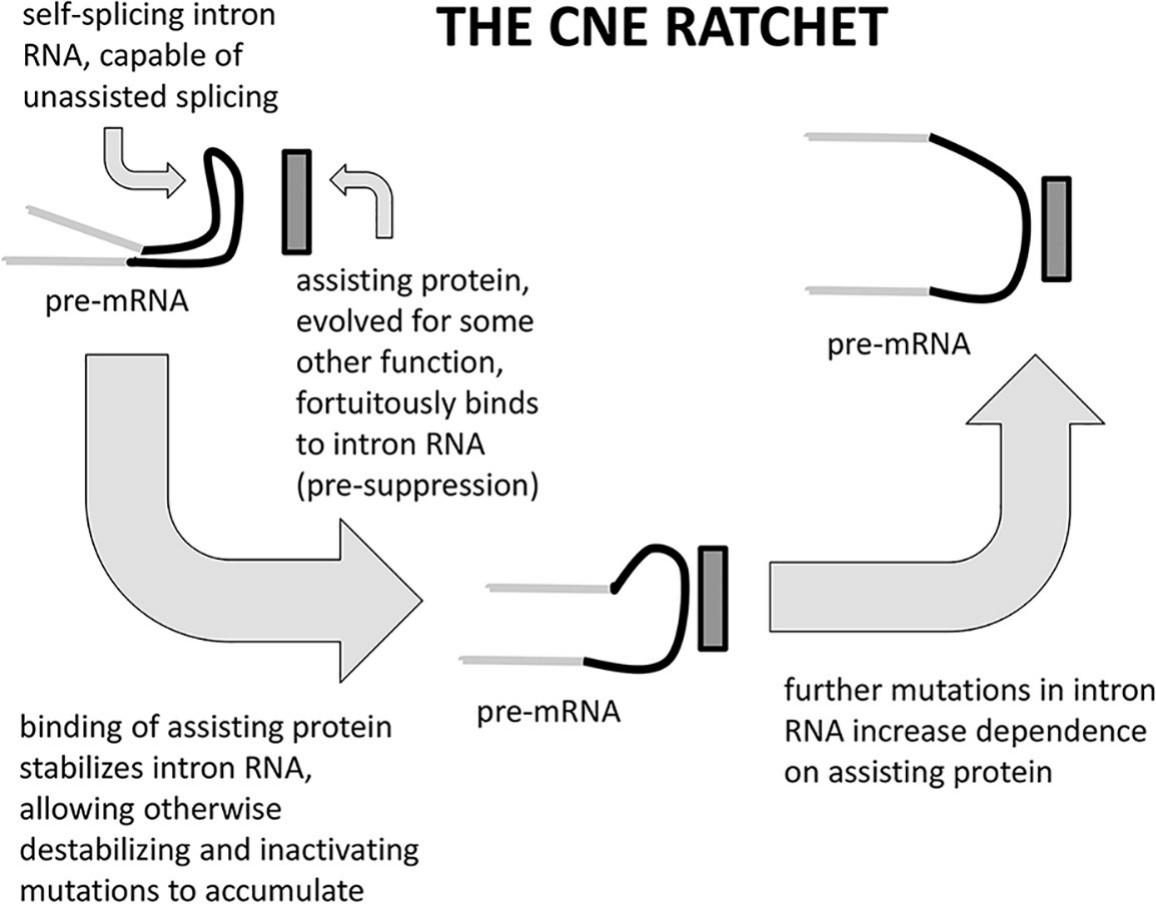
A simple case of Constructive Neutral Evolution. As Lambowitz and colleagues have shown [18], some strains of *Neurospora crassa* sport, in their mitochondrial rRNA genes, a group I intron which, because of its structure, is able to carry out a necessary interaction (self-splicing) without assistance of any protein. But in other strains, an unrelated protein fortuitously binds to and stabilizes the intron RNA. Destabilizing mutations in the RNA’s structure that would render it incapable of self-splicing without the bound protein are permitted ("pre-suppressed"). Such mutations are occasionally fixed by drift, and when more than one such mutation is possible, it is rare to reverse them all. Dependence on an assisting protein, initially a "pre-suppressor" of such mutations, is effectively locked-in.

CNE happens through molecular co-evolution. Two or more components (or two parts of the same component) fortuitously interact (directly or indirectly), allowing for the fixation of mutations in the first that would have been deleterious if it weren’t for this fortuitous interaction, now rendering that interaction, and the second component, necessary for the continued “functioning” of the first. We see such *pre-suppression* as a more likely evolutionary path for the evolution of dependency than *suppression*, because the latter entails a transient period of reduced fitness preceding the acquisition of the suppressing activity. (Suppression in experimental genetics is the relief of a deleterious mutant phenotype by a second mutation, often in a different gene.) Arlin Stoltzfus [17] described pre-suppression in terms of “excess capacity”, one component (the pre-suppressor) already having the potential to complement or buffer otherwise deleterious mutations in the other. These mutations then (assuming several potential sites for them) inevitably do occur, initiating a ratchet like process that makes reversal improbable (Fig 1).

CNE clearly applies to situations in which proteins have the ability (as an excess capacity) to stabilize RNAs or other proteins that might otherwise be rendered “dysfunctional” by mutation (Fig 1). Some of us have suggested that much of the complexity of the eukaryotic spliceosome, which arose (it is thought) as a self-splicing RNA but now requires the services of scores of proteins, can be explained in this way [19,20]. Situations in which a component B is necessary to counteract the activity of component A, but both can be deleted without consequences (e.g. toxin-antitoxin pairs), are also examples of CNE. We might similarly explain the evolution of hetero-oligomeric protein complexes from homo-oligomeric antecedents, or functional non-coding RNAs from a vast sea of junk RNA [19–24]. The feature that all such exemplary dependencies share [25] is that they arose by a non-selective process (no incremental increase in fitness), but have since been maintained by natural selection against loss (purifying selection). At least, we are assuming that components of these systems can vary and when such variation negatively affects the neutrally evolved interaction, the result would have negative impact on fitness.

We see all such instances, in which purifying selection explains the maintenance of traits that did not arise through positive or directional selection for the same effect, as importantly distinct from cases in which positive or directional selection gradually shaped the trait’s structure and function. In the former sorts of cases there is no need for incremental increases in fitness in the way that Maynard Smith describes hearts as having gotten better at pumping blood. Failure to recognize this distinction leads, at least in the current climate, to a kind of pan-adaptationism [26], where all traits at a level above that of neutral or nearly neutral variations in nucleotide sequence are assumed to be adaptations which have a “function” created by positive or directional selection. Thus, having gathered evidence that an element is under purifying selection, researchers will assume that it was positively selected for this (or some related) function, not even entertaining the possibility of neutral fixation or CNE.

In order to avoid this pitfall, our recommendation is to distinguish two kinds of selected effect functions that we call ***origin functions*** (which can only be inferred) and ***maintenance functions*** (which can often be experimentally confirmed) [4]. The former are always *adaptations* while the latter need never have been, though they affect *adaptedness* (contribute to fitness) and are under purifying selection. The distinction between adaptation and adaptedness is important. It is particularly obvious in the case of invasive species which may have features that promote their spread (and thus adaptedness) in new environments that were not adaptations in their previous settings, such as the robustness of zebra mussels in the ballasts of boats.

Neither origin nor maintenance functions, we think, should be sole possessor of the more general term “function”, because of the aforementioned concern about pan-adaptationism and because of the differing practices of evolutionary biologists in contrast to experimental biologists and genomicists. Evolutionary biology often focuses on adaptations that emerged and were modified by natural selection. This kind of investigation necessarily involves a reconstruction of ancestral environments which are often thought to be distinct from those encountered more recently. By contrast, experimental biologists and bioinformaticians identify functions by inferring (although seldom experimentally measuring) the effects of genetic manipulations on organismal fitness, or by looking for evidence of conservation within or among species. This last is evidence for past purifying selection but in principle tells us nothing about why or how the trait arose.

The intensity of the debate over the ENCODE project’s claim that 80.4% of the human genome comprises “functional elements” revealed (as we hinted in our third paragraph) how intertwined notions of function (in one or the other or both of the above senses) and selection are in the minds of biologists [3,4,6,8]. What that project failed to show was that fitness would be affected by deletion or inactivation of the vast majority of the “functional elements” or processes identified. At the same time, many of those elements showed little or no conservation across species. Hence, neither origin nor maintenance functions were experimentally demonstrated and this could only be explained by the assumption that such demonstration was considered unnecessary. That is, by the unacknowledged pan-adaptationism we seek here to expose and avoid. Some recognition of how entities originate, given the evolutionary dynamics of multicellular eukaryotes, could have helped ENCODE researchers to develop proper null models that would predict the existence of entities which, although functional according to the highly permissive CR definition, in fact have neither maintenance nor origin functions. Unless the notion of function is to be completely divorced from selection (as some proponents of CR “function” advise [27]), simply demonstrating that differences in “functional elements” cash out as differences in phenotype is not enough. There are wide differences in human phenotypes, as any quick look around any crowded room will show. But the majority of these traits probably have no bearing on survival or reproduction. If we embrace the CR definition and say that the function of genes for hair or eye color are to make the authors of this paper look different from each other, we are a long way from the definition of “function” endorsed by Maynard Smith (origin function), or the one that guides much of the research in molecular biology and bioinformatics (maintenance function).

To summarize, a trait’s selected effect can be an origin function or a maintenance function and we argue that authors should be clear about what they mean. In particular, it cannot be assumed without evidence that every entity has a maintenance function. Positive evidence for this claim (e.g. conservation) is required. Moreover, having provided evidence for a maintenance function it does not thereby follow that the entity also has an origin function. This is the main lesson to be drawn from CNE and other such examples. Occasionally an entity may have both origin and maintenance functions (the same or different), but sometimes it will have only one, and sometimes it might have neither [2]. How the traits of organisms are distributed among these categories is arguably biology’s deepest unsolved question, bringing the distinction between origin and maintenance functions back to the center, where it belongs.
